# Incompatibilities of Heroin and Heroin Hydrochloride

**Published:** 1901-01

**Authors:** 


### Abstracts.

### INCOMPATIBILITIES OF HEROIN AND HEROIN HYDROCHLORIDE.

Dr. Wesenberg (*Pharmaceutische Zeitung*) says that heroin and heroin hydrochloride form an essential part of so many formulæ for the relief of cough, dyspnea, and pains in the treatment of respiratory affections that it is important to determine in what combination they will prove most effective, and what are their incompatibilities. Owing to the insolubility of heroin in watery solutions it is necessary to add a few drops of some acid, acetic or hydrochloric, in order to effect its solution. This can be entirely obviated by using the hydrochloride, which is freely soluble. The only incompatibilities of heroin and the hydrochloride worthy of special mention are the alkalies, such as bicarbonate of sodium and carbonate of ammonium. On the other hand, salts of neutral reaction, such as iodide of potassium or chloride of ammonium may be used in the same mixture, and this also applies to acid salts, such as the hypophosphites or acid phosphates. The vegetable expectorants, as ipecac, senega, squill, and sanguinaria, are entirely compatible with heroin and its hydrochloride. Although many physicians employ heroin without admixture very desirable results have been reported from combinations with iodide of potassium, chloride of ammonium, and the vegetable expectorants, according to the indications present in particular cases. A word as to the dosage of heroin and heroin hydrochloride may be of interest here. The large does at first recommended at the time of the introduction of heroin are no longer preferred by the majority of authors, the average dose ranging from 1-24 to 1-12 grain in adults, and 1-120 to 1-60 grain in children. It is advisable not to employ larger doses until the smaller ones have been given a trial. Furthermore, many physicians now resort to the hypodermatic use of heroin hydrochloride in cases in which it is desirable to obtain an immediate effect, and especially in the treatment of spasmodic conditions, such as asthma, care being taken in the preparation of solutions not to add the drug until the water has partially cooled.

